# Natural image restoration based on multi-scale group sparsity residual constraints

**DOI:** 10.3389/fnins.2023.1293161

**Published:** 2023-11-06

**Authors:** Wan Ning, Dong Sun, Qingwei Gao, Yixiang Lu, De Zhu

**Affiliations:** Anhui Engineering Laboratory of Human-Robot Integration System and Intelligent Equipment, Key Laboratory of Intelligent Computing and Signal Processing of Ministry of Education, School of Electrical Engineering and Automation, Anhui University, Hefei, China

**Keywords:** image restoration, group sparsity residual, low-rank regularization, multi-scale, non-local self-similarity (NSS)

## Abstract

The Group Sparse Representation (GSR) model shows excellent potential in various image restoration tasks. In this study, we propose a novel Multi-Scale Group Sparse Residual Constraint Model (MS-GSRC) which can be applied to various inverse problems, including denoising, inpainting, and compressed sensing (CS). Our new method involves the following three steps: (1) finding similar patches with an overlapping scheme for the input degraded image using a multi-scale strategy, (2) performing a group sparse coding on these patches with low-rank constraints to get an initial representation vector, and (3) under the Bayesian maximum a posteriori (MAP) restoration framework, we adopt an alternating minimization scheme to solve the corresponding equation and reconstruct the target image finally. Simulation experiments demonstrate that our proposed model outperforms in terms of both objective image quality and subjective visual quality compared to several state-of-the-art methods.

## 1. Introduction

Unsuitable equipment and other disturbances unavoidably contribute noise in the target images. Image denoising is a crucial area of image processing and has attracted much attention from scholars in related fields recently. Digital image denoising techniques have a wide range of uses, involving disciplines of medicine and industry, and also in spectral images for weather forecasting, remote sensing images, and so on. Taking image denoising as a basis, the method can be introduced to more image restoration problems and be useful in more fields (Buades et al., 2005; Osher et al., 2005; Elad and Aharon, 2006; Zoran and Weiss, 2011; Gu et al., 2014; Zhang et al., 2014b; Liu et al., 2017; Keshavarzian et al., 2019; Ou et al., 2020; Zha et al., 2020a, 2022; Jon et al., 2021). This task aims to generate a latent image ***x*** from the degraded version ***y***. The process modeling can be depicted as


(1) $$\begin{array}{l} y=Hx+n \end{array}$$

Where ***H*** is an irreversible linear operator in matrix form and ***n*** is the additive white Gaussian noise vector. By requiring ***H***, Eq.(1) can be converted to diverse image restoration problems. For example, Eq.(1) represents the image denoising problem if ***H*** is an identity (Elad and Aharon, 2006; Ou et al., 2020); Eq.(1) denotes the image inpainting problem if ***H*** is a mask (Liu et al., 2017; Zha et al., 2020a); and Eq.(1) stands for the image CS problem if ***H*** is an undersampled random projection matrix (Keshavarzian et al., 2019; Zha et al., 2022). We concentrate on image denoising, inpainting, and CS challenges in this article.

Given that the problem always ill-posed, it is common to use image priors to regularize the model so as to gain excellent restored images. Namely, the Maximum A Posteriori (MAP) approach allows for the image restoration problem to be formulated as a mathematical equation to address the minimization problem:


(2) $$\begin{array}{l} \hat{x}=\underset{x}{\min}\frac{1}{2}\|y-Hx{\|}_{2}^{2}+\text{λ}R\left(x\right) \end{array}$$

The former is the data-fidelity term and the latter is the image prior constraint term. The weights between these two terms are regulated by the parameter λ. After establishing the mathematical model, we conceived an optimization algorithm to address various image restoration problems. The method yields a reconstructed image that approximates a clean image after several iterations.

Numerous image prior models have been put forward in earlier studies, mainly classified into local smoothness (Rudin et al., 1992; Osher et al., 2005; Dey et al., 2006), non-local self-similarity (Fazel et al., 2001; Buades et al., 2005; Gu et al., 2014), and sparsity (Zhang et al., 2014b; Ou et al., 2020, 2022a). Yet, the curse of dimensionality makes it difficult to construct a global model for the entire image. Therefore, the approach of building patch priors has become popular in recent years for its efficiency and convenience.

Sparse representation is one of the most representative patch-based priors. Elad and Aharon (2006) proposed K-SVD (K-Singular Value Decomposition) which is a pioneering work in applying sparse coding to image denoising. NSS is another crucial prior information widely used. Buades et al. (2005) proposed the first model using NSS for image denoising. In addition, the high correlation between patches leading to the data matrix of a clean image is as often low-rank. Related studies mainly fall into two categories: low-rank matrix factorization (LRMF) (Srebro and Jaakkola, 2003; Buades et al., 2005) and the Nuclear Norm Minimization (NNM) (Fazel et al., 2001; Hu et al., 2012). NNM is the more popular one in most cases. Gu et al. presented the Weighted Nuclear Norm Minimisation model (WNNM) (Gu et al., 2014) which dramatically enhances the flexibility of NNM, and it remains among most widespread image denoising methods. Apart from this, RRC (Zha et al., 2019), which makes use of low-rank residuals for modeling, has also achieved good quality in various image restoration problems.

Some studies have combined image sparsity and self-similarity to modeling, and these algorithms have shown great potential in image restoration research. For instance, in the study by Dabov et al. (2007), BM3D applies NSS to cluster patches before collaborative filtering, which is a benchmark method in the current area of image denoising. Both NCSR (Dong et al., 2012b) and GSR (Zhang et al., 2014b) use the NSS property to aggregate image patches into groups, and then perform sparse coding on the self-similar groups. Mairal J et al. devised the LSSC (Mairal et al., 2009) to force all self-similar groups to be imposed with the same dictionary. Zha et al. (2017) designed an efficient GSRC model that converts the task of image denoising into one of minimizing group sparse residuals. In addition, Zha et al. (2020a) also proposed a GSRC-NLP model with a better image restoration result based on the above.

Another groundbreaking patch-based image recovery method is Expected Patch Log Likelihood (EPLL) (Zoran and Weiss, 2011) which restores images by learning a Gaussian mixture model(GMM). Later on, Zoran et al. introduces a multi-scale EPLL (Papyan and Elad, 2015) model, which can improve the performance of image restoration further. Subsequently, image denoising methods using external GMM priors have been widely used. Most of the relevant studies have combined external GMM with internal NSS for modeling, such as Xu et al. (2015) proposed PGPD, Chen et al. (2015) proposed PCLR, and Zha et al. (2020b) proposed SNSS.

In addition to the above methods, deep convolutional neural networks (CNNs) (Zhang et al., 2017; Zhang and Ghanem, 2018) is an emerging approach in recent years, but it requires learning in an external database before restoring damaged images.

It is not comprehensive to only consider the sparsity or low-rankness property of the image. Hence, with the aim of obtaining a higher-quality restored image, our study uses the low-rank property of similar groups as a constraint in combination with sparsity to design the model. Furthermore, based on the NSS property, we can not only find similar patches for a specified patch on a single scale image but also extend the search window to multi-scales. Finally, we propose a novel Multi-scale Group Sparsity Residual Constraint (MS-GSRC) model with the following innovations:

1. We propose a novel MS-GSRC model that provides a simple yet effective approach for image restoration: find neighbor patches with an overlapping scheme for the input degraded image using a multi-scale strategy and perform a group sparse coding on these similar patches with a low-rank constraint.
2. An alternating minimization mechanism with an automatically tuned parameter scheme is applied to our proposed model, which guarantees a closed-form solution at each step.
3. Our proposed MS-GSRC model is validated on three tasks: denoising, inpainting, and compressed sensing. The model performs competitive in both objective image quality and subjective visual quality compared to several state-of-the-art image restoration methods.

The remainder of this article is as follows: In Section 2, after the brief overview of the GSRC framework and LR methods, we introduce a novel MS-GSRC model. Section 3 adopts an alternating minimization scheme with self-adjustable parameters to resolve our proposed algorithm. Section 4 lists extensive experimental results that prove the feasibility of our model. Conclusion is presented in Section 5.

## 2. Models

In this part, we briefly review some relevant knowledge and present our new model.

### 2.1. Group-based sparse representation

Principles of the GSR model can be described as follows: divide the image into many overlapping patches, find self-similarity groups for each image patch using the NSS property, perform sparse coding for each self-similarity group, and finally reconstruct the image (Dong et al., 2012b; Zha et al., 2020a; Ou et al., 2022a).

Specifically, the image ***x*** ∈ ℝ^*M*^ is divided into *m* overlapping patches ${\left\{x_{i}\right\}}_{i=1}^{m}$, where $x_{i}\in {\mathbb{R}}^{n\times n}$. Next, for each overlapping patch ***x*****_*i*_**, we use the K-Nearest Neighbor classification (KNN) algorithm (Keller et al., 1985; Xie et al., 2016) to select *k* neighbor patches from a *W*×*W* search window to form the group ***K*****_*i*_**. Subsequently, stack all ***K*****_*i*_** into a data matrix $X_{i}\in {\mathbb{R}}^{n\times k}$; this matrix contains each element of ***K*****_*i*_** as its column, i.e., ***X*****_*i*_** = {*x*_*i*, 1_, *x*_*i*, 2_…*x*_*i, k*_}, where ${\left\{x_{i,j}\right\}}_{j=1}^{k}$ denotes the k-th similar patch of the k-th group. Each similarity group ***X*****_*i*_** is represented sparsely as $\hat{X_{i}}=D{\hat{B}}_{i}$, where ***D*****_*i*_** denotes the dictionary.

Nevertheless, solving the 0-norm minimization problem is NP-hard, so for the ease of making the solution, The sparse code ${\hat{B}}_{i}$ is obtained from the following equation (Zhang et al., 2014b):


(3) $$\begin{array}{l} {\hat{B}}_{i}=\underset{B_{i}}{\min}\left(\frac{1}{2}\|X_{i}-D_{i}B_{i}{\|}_{F}^{2}+\text{λ}\|B_{i}{\|}_{1}\right)\;\forall i \end{array}$$

It is well-known that clean images ***x*** are unavailable in image restoration problems. Thus, we replace ***x*** with degenerate images ***y*** ∈ ℝ^*M* × *M*^. Eq.(3) can be transformed into the problem of recovering the group sparse code ***A***_*i*_ from ***Y*****_*i*_**:


(4) $$\begin{array}{l} {Â}_{i}=\underset{A_{i}}{\min}\left(\frac{1}{2}\|Y_{i}-D_{i}A_{i}{\|}_{F}^{2}+\text{λ}\|A_{i}{\|}_{1}\right)\;\forall i \end{array}$$

The restored ***X*****_*i*_** is obtained by $\hat{X_{i}}=D_{i}\hat{A_{i}}$, and the final complete image ***X*** can be gained by simple averaging ${\left\{X_{i}\right\}}_{i=1}^{m}$.

### 2.2. Group sparsity residual constraint

After observing the GSR model, it is clear that the closer the computed ***A*** approximates to ***B***, the better the quality of the final restoration image. Consequently, the following definition of the group sparsity residual constraint (GSRC) (Zha et al., 2017) is given: ***R*** = ***A*** − ***B***. Then, Eq.(4) for solving the group sparse coefficients ***A*****_*i*_** can be converted into:


(5) $$\begin{array}{l} {Â}_{i}=\underset{A_{i}}{\min}\left(\frac{1}{2}\|Y_{i}-D_{i}A_{i}{\|}_{F}^{2}+\text{λ}\|A_{i}-B_{i}{\|}_{1}\right)\;\forall i \end{array}$$

This model uses BM3D to restore the the degenerate observation ***y*** to the image ***z***. Moreover, ***z*** can be viewed as a good approximation of the target ***x*** considering BM3D has an excellent denoising performance. Thus, the group sparsity coefficients ***B*****_*i*_** can be obtained from ***z***. In the study by Zha et al. (2020a), GSRC-NLP uses NLP before constraining the input image.

### 2.3. Low-rank approximation

According to Gu et al. (2014), Zha et al. (2019), and Zha et al. (2020b), it can be found that NNM is a popular low-rank approximations methods. For ***X***, define the i-th singular value as *σ*_*i*_(***x***), and the nuclear norm as ‖***X***‖_*_ = Σ_*i*_*σ*_*i*_(***x***). The specific solution for ***X*** is:


(6) $$\begin{array}{l} \hat{X}=\underset{X}{\min}\|Y-X{\|}_{F}^{2}+\|X{\|}_{*}\;\forall i \end{array}$$

Equation (6) yields a simple solution: $\hat{X}=US_{\tau }V^{T}$, where **Ŷ** = ***U***Σ***V*****^*T*^** is the SVD for ***Y*** and ***S*****_τ_**(Σ) is a soft-thresholding (Cai et al., 2010) function. Namely, ***S*****_τ_(Σ)_*ii*_** = *max***(Σ_*ii*_ − τ, 0)**, where **Σ_*ii*_** is the diagonal element of **Σ**.

### 2.4. Multi-scale GSRC

The established GSRC model has performed well in image denoising, but it requires additional pre-processing of degraded images for obtaining the group sparsity coefficients ***B***. Thus, we combine group sparsity and low-rank property to build a model. Furthermore, the GSRC model only focuses on a single scale. However, it is evident that NSS can appear not only on the original scale of an image but also on a coarse scale, so we can find neighbor patches for the original image patch at multi-scales (Yair and Michaeli, 2018; Ou et al., 2022a,b). The specific steps of our proposed new Multi-Scale Group Sparse Residual Constraint (MS-GSRC) model are as follows:

(a) First, we use KNN to find a specified number of similar patches from both the original scale and scaled-down version for the overlapping patches of the input image.

(b) Then, these similar patches are stacked separately into groups.

(c) Next, the low-rank constraint is imposed on each group to obtain good group sparsity coefficients ***B*****_*i*_**.

(d) After estimating the group sparsity coefficients ***A*****_*i*_** by using the group sparsity residuals ***R*****_*i*_**, each group was recovered in sequence.

(e) Finally, we select the patch belonging to the original image from each group, and aggregate the complete image by simple averaging.

We propose the following constraint function:


(7) $$\begin{array}{r} \hat{x}=\underset{x}{\min}\frac{1}{2\sigma_{n}^{2}}\|y-Hx{\|}_{2}^{2}+\frac{1}{2\mu }\overset{m}{\underset{i=1}{\sum }}\|R_{i}x^{MS}\\ -D_{i}A_{i}{\|}_{F}^{2}+\text{λ}\overset{m}{\underset{i=1}{\sum }}\|A_{i}-B_{i}{\|}_{1}\;\forall i \end{array}$$

$R_{i}x^{MS}$ is a multi-scale similarity group, which is a matrix with ***k*** nearest neighbor patches matched for each original image patch. These similar patches are derived from both the original and coarse scales of the image. The window size is *W* × *W* in the original scale, and it isχ*W* × χ*W* in the other scale images, where **χ** indicates the scale factor (0 < **χ** < 1). **χ** will be set to different values in different experiments.

**Figure 1:**
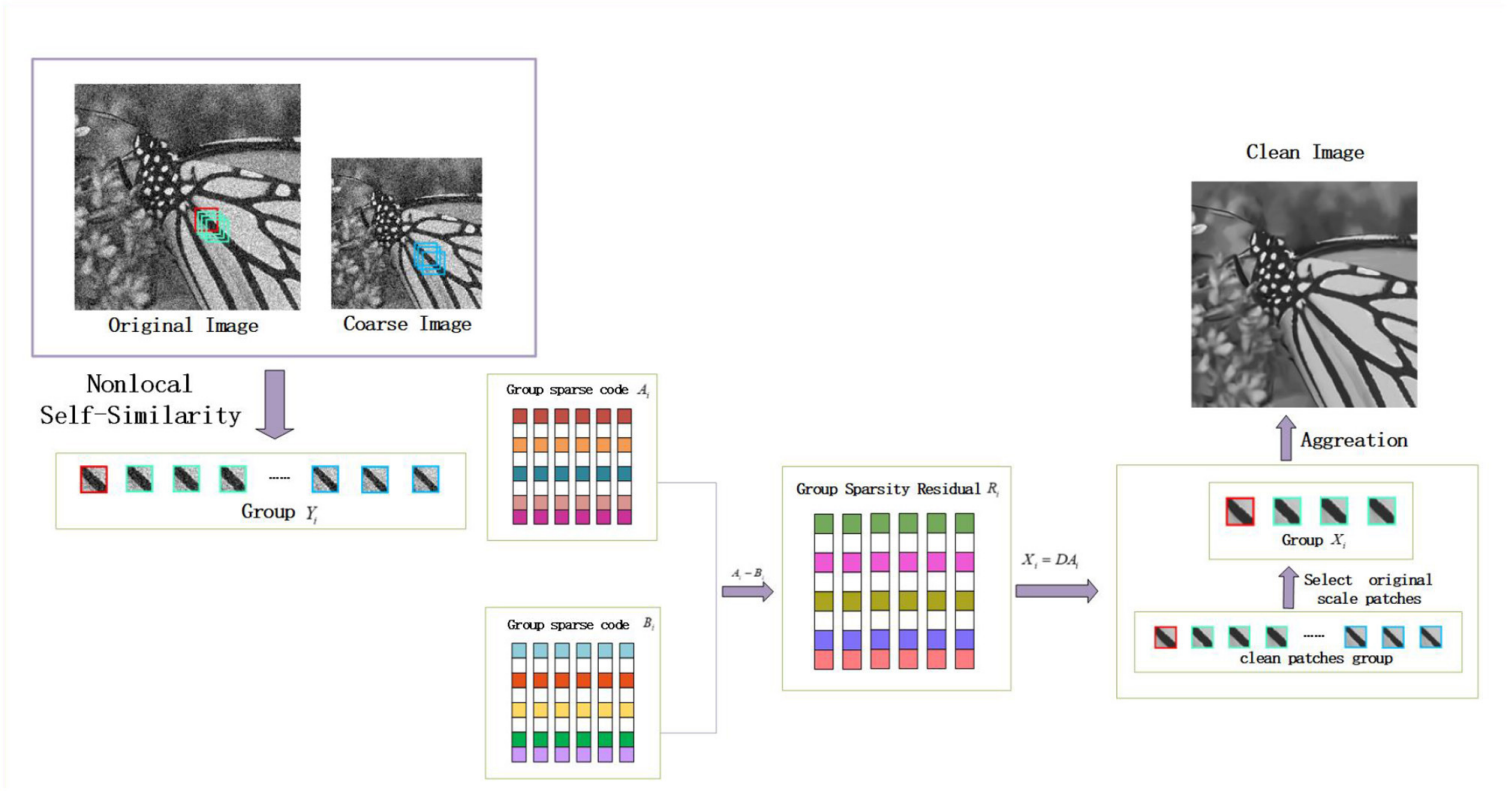
Flowchart of the proposed MS-GSRC for image denoising.

For image denoising, for example, the flowchart of MS-GSRC model is shown in Figure 1.

## 3. Algorithm for image restoration

This section is a detailed analysis of our proposed MS-GSRC model. The solution of this algorithm is obtained using an alternating minimization method whose parameter is self-adjustment.

First, we divide Eq.(7) into three sub-problems:

### 3.1. ***A*****_*i*_** sub-problem

Given ***x*** and ***D*****_*i*_**, we get a sub-problem of ***A*****_*i*_**:


(8) $$\begin{array}{l} {Â}_{i}=\underset{A_{i}}{\min}\overset{n}{\underset{i=1}{\sum }}\frac{1}{2\mu }\|R_{i}x^{MS}-D_{i}A_{i}{\|}_{F}^{2}+\text{λ}\|A_{i}-B_{i}{\|}_{1}\\ \;=\underset{A_{i}}{\min}\overset{m}{\underset{i=1}{\sum }}\|P_{i}-A_{i}{\|}_{F}^{2}+2\text{λ}\mu \|A_{i}-B_{i}{\|}_{1}\\ \;=\underset{\alpha_{i}}{\min}\overset{m}{\underset{i=1}{\sum }}\|p_{i}-\alpha_{i}{\|}_{F}^{2}+2\text{λ}\mu \|\alpha_{i}-\beta_{i}{\|}_{1} \end{array}$$

where $P_{i}=D_{i}^{-1}R_{i}x^{MS}$, ***α*****_*i*_**, ***β*****_*i*_**, ***p*****_*i*_** stand for the vector representations of ***A*****_*i*_**, ***B*****_*i*_**, and ***P*****_*i*_**, respectively. ***D*****_*i*_** is a dictionary, A crucial step for solving the ***A*****_*i*_** problem is to design an efficient ***D*****_*i*_**. The restored image is prone to visual artifacts (Lu et al., 2013) if learning the over-complete dictionary. To reduce this terrible phenomenon, we choose to adopt principal component analysis (PCA) (Abdi and Williams, 2010) for learning the dictionary ***D*****_*i*_** in this study because PCA is more robust and adjustable.

Equation (8) can be deduced as a closed-form solution:


(9) $$\begin{array}{l} {Â}_{i}=Soft\left({\tilde{p}}_{i}-{\tilde{\beta }}_{i},2\text{λ}\mu \right)+{\tilde{\beta }}_{i}\;\forall i \end{array}$$

***Soft*****(·)** represents the soft-thresholding operator.

Since ***x*** is an unknown target image, it is impossible to gain the group sparse coefficients ***B*****_*i*_** directly. Consequently, we must utilize methods to gain an approximation value.

The introduction of low-rank constraints into the model is a practical approach. After applying LR constraints to the ***Y*****_*i*_** group, we can obtain a matrix ***S*****_*i*_**. The clean group sparsity coefficients ***B*****_*i*_** can be computed from ***S*****_*i*_**. It is easy to derive the following equation:


(10) $$\begin{array}{l} \|Y_{i}-S_{i}{\|}_{F}^{2}=\|A_{i}-B_{i}{\|}_{F}^{2} \end{array}$$

So,we can obtain


$$\begin{array}{l} \begin{array}{c} {Ŝ}_{i}=\arg\underset{S_{i}}{\min}\frac{1}{2}\|Y_{i}-S_{i}{\|}_{F}^{2}+\theta \|S_{i}{\|}_{*}\\ \Leftrightarrow {\hat{B}}_{i}=\arg\underset{B_{i}}{\min}=\frac{1}{2}\|A_{i}-B_{i}{\|}_{F}^{2}+\theta \|B_{i}{\|}_{*} \end{array} \end{array}$$

where ‖***B*****_*i*_**‖_*_ = Σ_*j*_δ_*i, j*_, ${\left\{\delta_{i,j}\right\}}_{j=1}^{s}$ are singular value of matrix. Apparently, we are able to get a closed-form solution for ***B***_*i*_:


(11) $$\begin{array}{l} {\hat{B}}_{i}=U_{i}soft\left({\text{Δ}}_{i},\theta \right)V_{i}^{T}\;\forall i \end{array}$$

where $A_{i}=U_{i}{\text{Δ}}_{i}V_{i}^{T}$ is the SVD for ***A*****_*i*_** and **Δ_*i*_** is the diagonal element of the singular matrix.

### 3.2. ***x*** sub-problem

Given ***A*****_*i*_** and ***D*****_*i*_**, subproblem of ***x*** in Eq.(7) turns into:


(12) $$\begin{array}{l} \hat{x}=\underset{x}{\min}\frac{1}{2\sigma_{n}^{2}}\|y-Hx{\|}_{2}^{2}+\frac{1}{2\mu }\overset{m}{\underset{i=1}{\sum }}\|R_{i}x^{MS}-D_{i}A_{i}{\|}_{F}^{2} \end{array}$$

Clearly, Eq.(13) is a quadratic optimization equation. We adopt Alternate Directional Multiplication Method (ADMM) (Boyd et al., 2011) to simplify the optimization process.

First, we bring in an auxiliary variable ***s***** = *x*^*MS*^**, and Eq.(13) can be converted into an equivalence equation:


(13) $$\begin{array}{l} \langle \hat{x},ŝ\rangle =\underset{x,s}{\min}\frac{1}{2\sigma_{n}^{2}}\|y-Hx{\|}_{2}^{2}+\frac{1}{2\mu }\overset{m}{\underset{i=1}{\sum }}\|R_{i}s-D_{i}A_{i}{\|}_{F}^{2} \end{array}$$

By observing Eq.(14), it is plain that this equation has three unknown variables requiring solutions. Thus, we decompose Eq.(14) into three iterative processes. In the t-th iteration:


(14) $$\begin{array}{l} \;{\hat{x}}^{t+1}=\underset{x}{\min}\frac{1}{2\sigma_{n}^{2}}\|y-Hx{\|}_{2}^{2}+\frac{1}{2\zeta }\|x-s^{t}-c^{t}{\|}_{2}^{2} \end{array}$$

(15) $$\begin{array}{l} \;{ŝ}^{t+1}=\underset{s}{\min}\frac{1}{2\mu }\sum_{i=1}^{m}\|R_{i}s-D_{i}A_{i}{\|}_{F}^{2}+\frac{1}{2\zeta }\|x^{t+1}-s-c^{t}{\|}_{2}^{2} \end{array}$$

(16) $$\begin{array}{l} \;c^{t+1}=c^{t}-\left(x^{t+1}-s^{t+1}\right) \end{array}$$

The parameter ***c*** indicates the Lagrangian multiplier. To make the derivation process look more concise, we omit ***t*** in the following formulation expression.

***Update***** *s*** : Given ***D*****_*i*_*A*_*i*_**, ***x***, and ***c***, ***s*** can be represented as a closed-form solution by Eq.(16), namely:


(17) $$\begin{array}{l} ŝ={\left(\mu I+\zeta \overset{m}{\underset{i=1}{\sum }}R_{i}^{T}R_{i}\right)}^{-1}\left(\mu x-\mu c+\zeta \overset{m}{\underset{i=1}{\sum }}R_{i}^{T}D_{i}A_{i}\right) \end{array}$$

Since ***I*** is a matrix of identities and $R_{i}^{T}R_{i}$ represents a diagonal matrix, $\left(\mu I+\zeta \overset{m}{\underset{i=1}{\sum }}R_{i}^{T}R_{i}\right)$ is positive. So the above formula is valid.

***Update***** *x*** : Given ***s*** and ***c***, Eq.(15) provides a solution to the variable ***x***:


(18) $$\begin{array}{l} \hat{x}={\left(\zeta H^{T}H+\sigma_{n}^{2}I\right)}^{-1}\left(\zeta H^{T}y+\sigma_{n}^{2}s+\sigma_{n}^{2}c\right) \end{array}$$

Notably, since ***H*** is an unstructured random projection matrix, the cost required to solve ***x*** using Eq.(19) directly is too high in CS problem. Hence, after setting step size γ and gradient direction *q*, we employ the gradient descent method (Ruder, 2016): $\hat{x}=x-\gamma q$ to rewrite Eq.(19) as:


(19) $$\begin{array}{l} \hat{x}=x-\gamma \left(\frac{1}{\sigma_{n}^{2}}\left(H^{T}Hx-H^{T}y\right)+\frac{1}{\zeta }\left(x-s-c\right)\right) \end{array}$$

In addition, it is recommended to compute ***H*****^*T*^*H*** and ***H*****^*T*^*y*** in advance to further enhance the algorithm efficiency.

### 3.3. Parameter settings

In the model we proposed above, there are four parameters (**μ**, **λ**, **θ**, **ζ**) requiring setting. Here, we set a strategy for the parameters that can be automatically adjusted in each iteration, which allows us to achieve more robust and accurate experimental results.

The noise standard deviation ***σ*****_*n*_** is automatically updated in each iteration (Osher et al., 2005):


(20) $$\begin{array}{l} \sigma_{e}^{t}=\omega \sqrt{\sigma_{n}^{2}-\|y-{\hat{x}}^{\left(t\right)}{\|}_{2}^{2}} \end{array}$$

Where ω represents a scaling factor, it is evident from Gu et al. (2014) and Chen et al. (2015) that this approach to regularize ***σ*****_*e*_** has been implemented in diverse models and has exhibited positive performance.

After setting ***σ*****_*e*_**, the value of μ is tuned to change in proportion to $\sigma_{e}^{2}$ (Zha et al., 2022):


(21) $$\begin{array}{l} \mu =\rho {\left(\sigma_{e}^{2}\right)}^{t} \end{array}$$

where ρ denotes a constant.

Moreover, the regularization parameters λ and θ represent the constraint penalties on sparsity and LR, respectively. Inspired by Dong et al. (2012a), they are adjusted in each iteration as follows:


(22) $$\begin{array}{l} {\text{λ}}^{\left(t\right)}=\frac{2\sqrt{2}\alpha {\left(\sigma_{e}^{t}\right)}^{2}}{m_{i}+\varepsilon }\;\theta^{\left(t\right)}=\frac{2\sqrt{2}\beta {\left(\sigma_{e}^{t}\right)}^{2}}{n_{i}+\epsilon } \end{array}$$

where ***m*****_*i*_** is the estimated standard variance of ***R*****_*i*_** and ***n*****_*i*_** stands for the estimated standard variance of **Δ_*i*_**. The ε and ϵ are two small constants to avoid zero divisors. *α* and *β* are set to two constants. Finally, parameter ζ is also set to a fixed constant.

The detailed procedure of the MS-GSRC algorithm is presented in Algorithm 1.

**Algorithm 1:** The MS-GSRC algorithm for image restoration.

**Require:** The observation ***y*** and the degradation operator ***H***.
1: Initialize ${\hat{x}}^{0}=y$ and parameters *m*, *n*, *W*, γ, ζ, *α*, *β*, χ, ε,ϵ and *Iter*.
2: **for** t=1:Iter **do**
3: Update parameters by Eq.(21-23);
4: divide ***x***^(*t*)^ into patches ${\left\{x_{i}\right\}}_{i=1}^{m}$.
5: **for** each ***x*****_*i*_** **do**
6: Construct multi-scale group $R_{i}x^{MS}$;
7: **end for**
8: **for** each group $R_{i}x^{MS}$ **do**
9: Construct dictionary ***D*****_*i*_** by ***P*****_*i*_** by using PCA;
10: Compute ***A*****_*i*_** by Eq.(9);
11: Compute ***B*****_*i*_** by Eq.(12);
12: **end for**
13: ADMM:
14: Initialize:***c*** = **0** and $s=\hat{x}$.
15: Compute ***s***^(*t*+1)^ by Eq.(18);
16: Compute ***c***^(*t*+1)^ by Eq.(17);
17: **if** H is an unnstructured random projection matrix **then**
18: Construct ***x***^(*t*+1)^ by Eq.(20);
19: **else**
20: Construct ***x***^(*t*+1)^ by Eq.(19);
21: **end if**
22: **end for**
23: Output:Restored image $\hat{x}$.

## 4. Experiences

In this chapter, extensive trial are conducted on image denoising, inpainting, and CS to verify that our proposed MS-GSRC model possesses better image restoration capabilities compared to some classical methods. To obtain intuitive comparison results, we set on two metrics: peak signal-to-noise ratio (PSNR) and structural self-similarity (SSIM) (Wang et al., 2004).

PSNR is commonly used to measure signal distortion. This parameter is calculated based on the gray scale values of the image pixels. Although sometimes the value of PSNR is not consistent with competent human perception, it remains an important reference evaluation metric. SSIM is a metric intended for assessing similarity between two images, which is an intuitive human standard for evaluating image quality.

If the degraded image is in color, we mainly recover the luminance channel due to the fact that variations in the luminance of color images are more easily perceived by the human eye.

**Figure 2:**
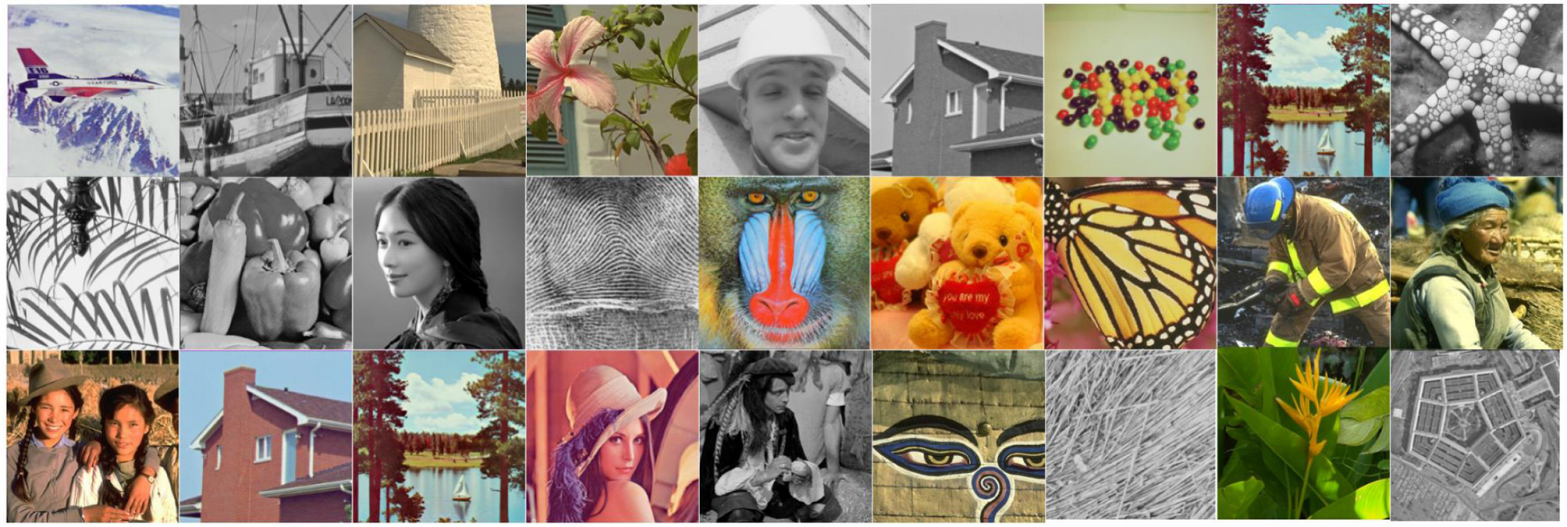
The 27 widely tested images for experiences.

The codes for all comparison algorithms used in this study are obtained from the original author's homepage and uses the given default parameters directly. For reasons of limited space, only a few images frequently used for testing are detailed list in Figure 2. In all tables, the data marked in red represent the best values.

### 4.1. Image denoising

First, we verify the performance of our MS-GSRC model on the image denoising task. The corresponding parameters are set as follows. We set the search window *W* × *W* to 30 × 30, the patch size $\sqrt{m}\times \sqrt{m}$ to 6 × 6, 7 × 7, 9 × 9 for *σ* ≤ 15, 15 < *σ* ≤ 30, and 30 < *σ* ≤ 75, with the number of neighbor patches *k* to 70, 110, 120 for *σ* ≤ 30, 30 < *σ* ≤ 50, 50 < *σ* ≤ 75, respectively. The parameters (*α*, *β*, ω, ζ) are set to (0.03, 1.75, 0.81, 0.085), (0.015, 1.8, 0.86, 0.07), (0.05, 2.2, 0.81, 0.12), (0.006, 2, 0.86, 0.05) for *σ* ≤ 15, 15 < *σ* ≤ 30, 30 < *σ* ≤ 50, 50 < *σ* ≤ 75. In addition, we set the multi-scale to [1,0.8], [1,0.85], and [1,0.9] for *σ* ≤ 15, 15 < *σ* ≤ 50, and 50 < *σ* ≤ 75, separately.

Our MS-GSRC method is compared with several recently proposed popular denoising methods and classical traditional denoising methods, including BM3D (Dabov et al., 2007), PGPD (Xu et al., 2015), WNNM (Gu et al., 2014), NCSR (Dong et al., 2012b), RRC (Zha et al., 2019), LGSR (Zha et al., 2022) and GSRC-NLP (Zha et al., 2020a). Of all the comparison methods, BM3D is a frequently adopted benchmarking method, NCSR, PGPD, and GSRC-NLP all use GSR as a prior, and WNNM and RRC exploit low-rankness knowledge. And LGSR combines GSR and LR. Besides, both GSRC-NLP and our proposed model use the GSRC framework. Taking 12 frequently used images as an example, Table 1 lists the PSNR and SSIM results for various denoising methods at different noise levels. It is observed that our proposed MS-GSRC method produced superior performance. Specifically, the average PSNR and SSIM we achieve are improved by (0.47 dB, 0.0149) compared to BM3D, (0.38 dB, 0.0107) compared to PGPD, (0.07 dB, 0.0032) compared to WWNM, (0.42 dB, 0.0149) compared to NCSR, (0.25 dB, 0.0066) compared to RRC, (0.1 dB, 0.0005) compared to LGSR, and (0.19 dB, 0.0054) compared to GSRC-NLP.

**Table 1:** PSNR (dB) and SSIM comparison of different methods for image denoising.

**Image**	**Airplane**	**Flower**	**Foreman**	**J.Bean**	**Lake**	**Leaves**	**Lena**	**Lin**	**Monarch**	**Starfish**	**Pentagon**	**Peppers**	**Average**
*σ* **= 15**													
BM3D	32.14	31.57	35.68	35.70	30.45	31.72	33.04	34.23	31.86	31.15	29.68	31.80	32.42
	0.9230	0.9074	0.9178	0.9693	0.9063	0.9648	0.9209	0.9243	0.9353	0.8958	0.8716	0.8764	0.9177
PGPD	32.31	31.85	35.51	35.65	30.67	32.02	33.13	34.16	32.23	31.31	29.72	31.78	32.53
	0.9193	0.9076	0.9140	0.9582	0.9086	0.9671	0.9185	0.9110	0.9362	0.9024	0.8724	0.8725	0.9156
WNNM	32.47	32.04	**35.88**	36.56	30.83	32.83	**33.34**	**34.47**	32.72	**31.83**	**30.06**	**32.03**	32.92
	0.9252	0.9132	0.9234	0.9735	0.9129	0.9735	0.9248	**0.9227**	0.9424	**0.9081**	0.8810	**0.8770**	0.9231
NCSR	**32.95**	31.77	35.52	**37.89**	**31.21**	32.16	33.04	34.27	32.31	31.46	29.93	31.86	32.86
	0.9201	0.9082	0.9189	**0.9782**	0.8965	0.9694	0.9192	0.9190	0.9401	0.9042	0.8779	0.8725	0.9187
RRC	32.38	31.81	35.71	36.16	30.70	32.55	33.23	34.31	32.61	31.50	29.72	31.85	32.71
	0.9248	0.9076	0.9225	0.9734	0.9091	0.9719	0.9233	0.9197	0.9435	0.8988	0.8693	0.8720	0.9197
LGSR	32.47	32.02	**35.88**	36.40	30.84	32.73	33.32	34.43	32.69	31.67	30.05	32.00	32.87
	0.9255	0.9127	**0.9244**	0.9751	0.9130	0.9732	**0.9249**	0.9222	0.9435	0.9040	0.8818	0.8753	0.9230
GSRC-NLP	32.37	31.97	35.84	36.10	30.76	32.61	33.23	34.33	32.59	31.55	29.96	31.96	32.77
	0.9240	0.9107	0.9235	0.9720	0.9107	0.9729	0.9234	0.9183	0.9422	0.9007	0.8764	0.8748	0.9208
OURS	32.56	**32.10**	35.80	36.69	30.93	**32.99**	33.33	34.46	**32.78**	**31.83**	30.03	**32.03**	**32.96**
	**0.9266**	**0.9141**	0.9229	0.9741	**0.9162**	**0.9745**	0.9245	0.9203	**0.9436**	0.9072	**0.8860**	0.8764	**0.9239**
*σ* **= 30**													
BM3D	28.49	27.97	32.75	31.97	26.74	27.81	29.46	30.95	28.36	27.65	26.41	28.66	28.94
	0.8642	0.8204	0.8779	0.9371	0.8256	0.9254	0.8590	0.8701	0.8808	0.8217	0.7492	0.8167	0.8540
PGPD	28.63	28.11	32.83	31.99	26.90	27.99	29.60	30.96	28.49	27.67	26.31	28.70	29.02
	0.8646	0.8213	0.8818	0.9317	0.8294	0.9300	0.8622	0.8606	0.8853	0.8277	0.7400	0.8164	0.8542
WNNM	28.75	28.34	**33.23**	32.50	27.02	28.61	29.72	31.07	28.91	28.07	**26.66**	**28.84**	29.31
	0.8698	0.8318	0.8892	0.9438	0.8355	0.9389	0.8670	0.8643	0.8926	0.8357	**0.7615**	0.8201	0.8625
NCSR	28.40	27.58	32.66	32.85	26.65	28.24	29.35	30.71	28.59	27.77	26.37	28.64	28.99
	0.8473	0.7704	0.8853	0.9468	0.7902	0.9377	0.8583	0.8669	0.8890	0.8304	0.7492	0.8153	0.8489
RRC	28.63	28.12	33.27	32.33	26.89	28.35	29.67	30.96	28.79	27.95	26.33	28.67	29.16
	0.8716	0.8240	0.8952	0.9482	0.8323	0.9366	0.8672	**0.8703**	0.8954	0.8304	0.7374	0.8184	0.8606
LGSR	28.76	28.30	33.36	32.32	27.05	28.48	29.78	30.96	28.87	28.02	26.58	28.77	29.27
	0.8749	0.8316	**0.8960**	**0.9491**	0.8378	0.9386	**0.8718**	0.8663	0.8952	0.8348	0.7541	0.8204	0.8642
GSRC-NLP	28.68	28.21	33.15	32.28	26.89	28.56	29.66	30.92	28.80	28.02	26.41	28.71	29.19
	0.8726	0.8262	0.8941	0.9482	0.8303	0.9401	0.8682	0.8647	0.8939	0.8313	0.7383	0.8186	0.8605
OURS	**28.85**	**28.38**	33.09	**32.63**	**27.10**	**28.90**	**29.79**	**31.13**	**28.97**	**28.23**	26.50	28.82	**29.37**
	**0.8767**	**0.8332**	0.8912	0.9470	**0.8418**	**0.9431**	0.8692	0.8680	**0.8957**	**0.8405**	0.7551	**0.8218**	**0.8653**
*σ* **= 50**													
BM3D	25.76	25.49	30.36	29.26	24.29	24.68	26.90	28.71	25.82	25.04	24.21	26.17	26.39
	0.7967	0.7311	0.8396	0.9038	0.7381	0.8639	0.7938	0.8200	0.8197	0.7377	0.6282	0.7548	0.7856
PGPD	25.98	25.63	30.45	29.20	24.49	25.03	27.15	28.79	26.00	25.11	24.17	26.31	26.53
	0.8059	0.7324	0.8410	0.8934	0.7483	0.8794	0.7990	0.8118	0.8269	0.7457	0.6206	0.7578	0.7885
WNNM	26.18	25.93	30.98	29.63	24.56	25.47	27.27	28.74	26.32	25.43	24.47	26.41	26.78
	0.8133	0.7502	0.8548	0.9098	0.7567	0.8926	0.8074	0.8138	0.8350	0.7596	0.6418	0.7630	0.7998
NCSR	25.63	25.31	30.41	29.24	24.15	24.94	26.94	28.23	25.73	25.06	23.92	26.04	26.30
	0.8066	0.7217	0.8559	0.9134	0.7420	0.8787	0.8009	0.8171	0.8252	0.7440	0.6058	0.7567	0.7890
RRC	26.13	25.72	30.87	29.38	24.48	25.30	27.17	28.51	26.22	25.34	24.21	26.23	26.63
	0.8171	0.7413	0.8611	0.9125	0.7571	0.8910	0.8073	0.8140	0.8361	0.7589	0.6162	0.7643	0.7981
LGSR	26.15	25.92	31.03	29.40	24.59	25.39	27.27	28.56	26.24	25.40	24.47	26.37	26.73
	**0.8212**	**0.7544**	**0.8637**	0.9141	0.7629	0.8930	**0.8140**	0.8171	0.8364	0.7616	0.6423	**0.7655**	0.8039
GSRC-NLP	26.17	25.76	30.77	29.58	24.44	25.66	27.06	28.60	26.25	25.36	24.24	26.32	26.69
	0.8201	0.7416	0.8610	**0.9166**	0.7492	0.8991	0.8014	0.8153	0.8297	0.7540	0.6125	0.7633	0.7970
OURS	**26.23**	**26.02**	**31.08**	**29.67**	**24.64**	**25.79**	**27.34**	**28.82**	**26.39**	**25.59**	**24.49**	**26.44**	**26.87**
	0.8209	0.7530	0.8605	0.9067	**0.7631**	**0.8991**	0.8110	**0.8188**	**0.8369**	**0.7663**	**0.6473**	**0.7665**	**0.8042**
*σ* **= 75**													
BM3D	23.99	23.82	28.07	27.22	22.63	22.49	25.17	26.96	23.91	23.27	22.59	24.43	24.55
	0.7331	0.6515	0.7880	0.8613	0.6636	0.8021	0.7310	0.7704	0.7557	0.6619	0.5240	0.6973	0.7200
PGPD	24.15	23.82	28.39	27.07	22.76	22.61	25.30	27.05	24.00	23.23	22.55	24.46	24.62
	0.7492	0.6468	0.7965	0.8503	0.6760	0.8121	0.7356	0.7669	0.7642	0.6638	0.5145	0.7026	0.7232
WNNM	24.25	24.07	28.95	27.42	22.76	23.06	25.52	26.91	24.31	22.84	24.45	23.47	24.84
	0.7601	0.6697	0.8133	0.8707	0.6850	0.8351	0.7514	0.7717	0.7754	0.5412	0.7035	0.6801	0.7381
NCSR	23.76	23.50	28.18	27.15	22.48	22.60	25.02	26.22	23.67	23.18	22.10	24.19	24.34
	0.7547	0.6409	0.8171	0.8792	0.6743	0.8234	0.7415	0.7730	0.7648	0.6685	0.4881	0.7073	0.7277
RRC	24.10	23.77	28.83	27.17	22.64	22.91	25.33	26.86	24.24	23.32	22.56	24.35	24.67
	0.7638	0.6499	0.8259	0.8749	0.6822	0.8377	0.7498	0.7729	0.7782	0.6741	0.5028	0.7172	0.7358
LGSR	24.25	24.14	29.10	27.37	22.74	23.09	**25.55**	26.97	24.31	23.43	**22.91**	24.56	24.87
	0.7709	**0.6772**	**0.8296**	0.8828	0.6836	0.8410	**0.7577**	**0.7839**	0.7794	0.6805	**0.5354**	0.7190	0.7451
GSRC-NLP	24.13	23.88	28.76	27.29	22.61	23.33	25.32	26.84	24.35	23.32	22.65	24.45	24.74
	0.7671	0.6614	0.8251	0.8796	0.6772	0.8512	0.7480	0.7806	0.7779	0.6712	0.5146	0.7179	0.7393
OURS	**24.32**	**24.19**	**29.11**	**27.62**	**22.80**	**23.49**	25.51	**27.24**	**24.48**	**23.56**	22.65	**24.64**	**24.97**
	**0.7721**	0.6677	0.8273	**0.8851**	**0.6916**	**0.8514**	0.7545	0.7873	**0.7807**	**0.6859**	0.5269	**0.7231**	**0.7461**

The data marked in red represent the best values.

**Figure 3:**
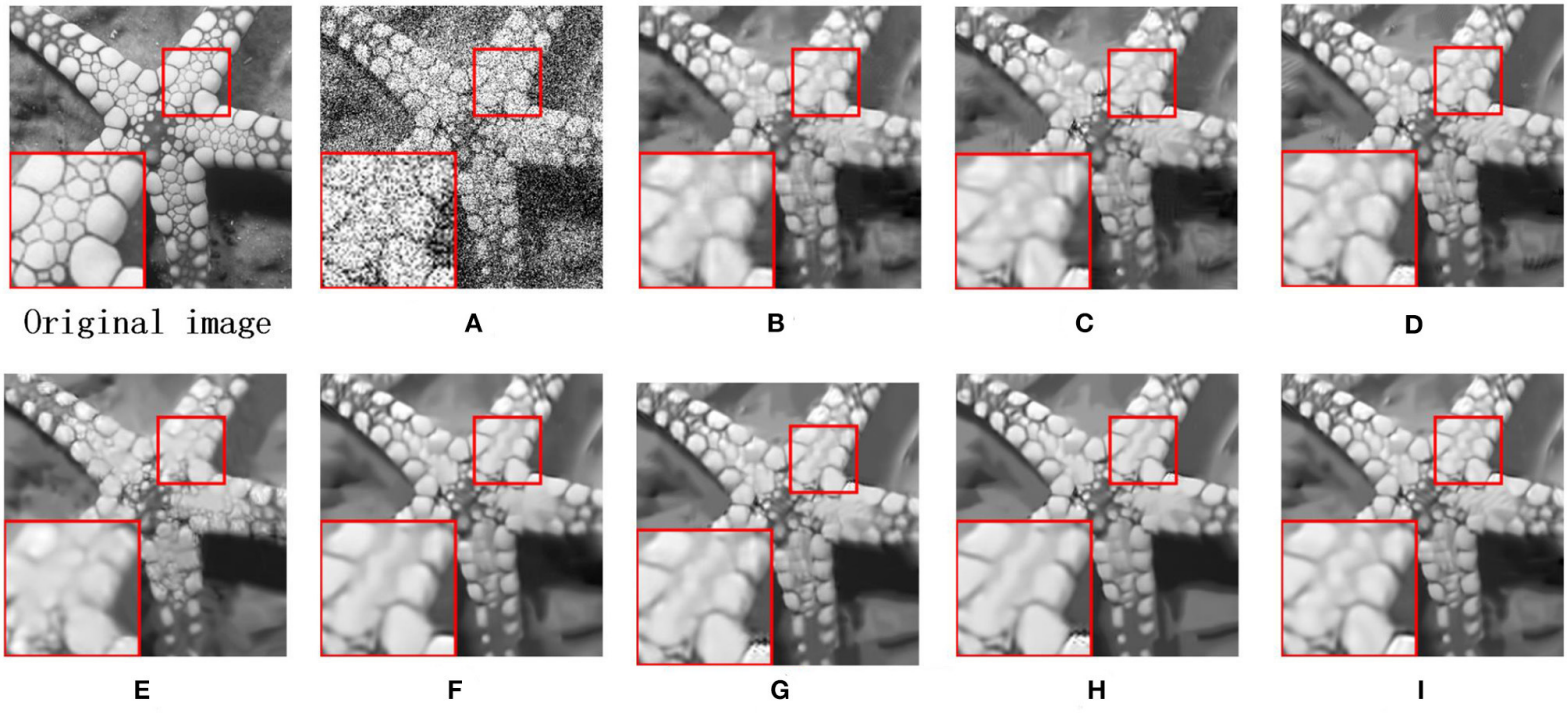
Denosing results on image starfish (*σ* = 75). **(A)** Noise image. **(B)** BM3D (PSNR = 23.27 dB and SSIM = 0.6619). **(C)** PGPD (PSNR = 23.23 dB and SSIM = 0.6638). **(D)** WNNM (PSNR = 22.84 dB and SSIM = 0.5412). **(E)** NSRC (PSNR = 23.18 dB and SSIM = 0.6685). **(F)** RRC (PSNR = 23.32 dB and SSIM = 0.6741). **(G)** LGSR (PSNR = 23.43 dB and SSIM = 0.6805). **(H)** GSRC-NLP (PSNR = 23.32 dB and SSIM = 0.6712). **(I)** OURS (PSNR = **23.56** dB and SSIM = **0.6859**).

**Figure 4:**
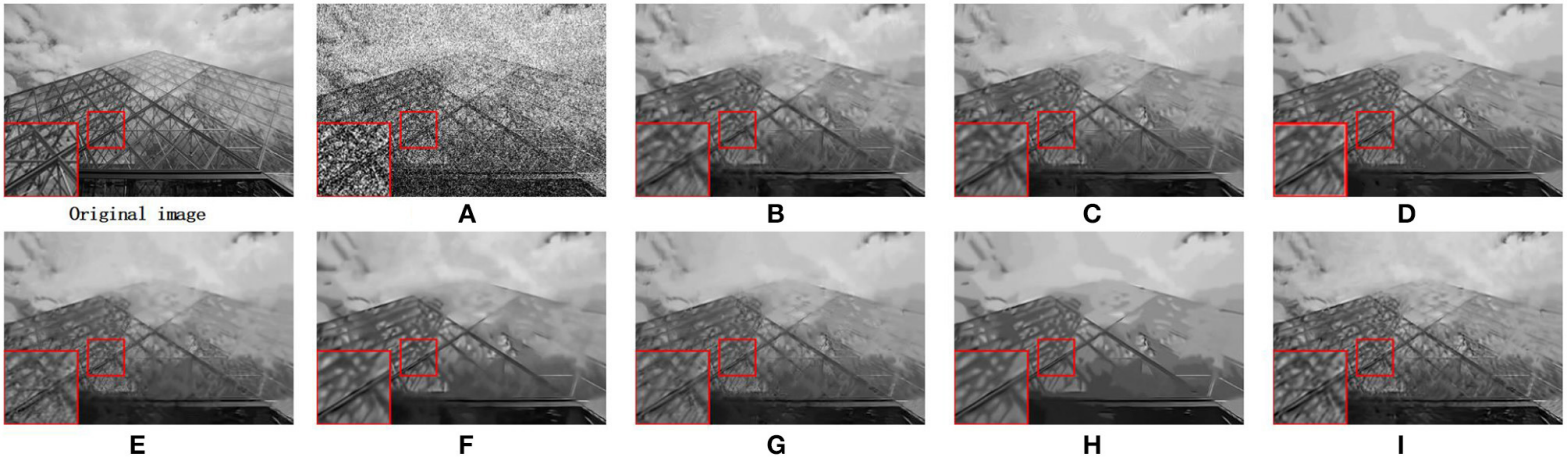
Denosing results on image 223061 (*σ* = 75). **(A)** Noise image. **(B)** BM3D (PSNR = 22.27 dB and SSIM = 0.5470). **(C)** PGPD (PSNR = 22.30 dB and SSIM = 0.5420). **(D)** WNNM (PSNR = **22.51** dB and SSIM = 0.5690). **(E)** NSRC (PSNR = 22.15 dB and SSIM = 0.5383). **(F)** RRC (PSNR = 22.22 dB and SSIM = 0.5351). **(G)** LGSR (PSNR = 22.32 dB and SSIM = 0.5545). **(H)** GSRC-NLP (PSNR = 22.13 dB and SSIM = 0.5313). **(I)** OURS (PSNR = 22.42 dB and SSIM = **0.5761**).

We also utilize the BSD68 dataset (Wang et al., 2004) to assess the denoising ability of all compared approaches. We can observe from Table 2 that the average PSNR gains obtained by our proposed MS-GSRC method in comparison to the BM3D, PGPD, WNNM, NCSR, RRC, GSRC-NLP, and LGSR methods are 0.24 dB, 0.16 dB, 0.02 dB, 0.27 dB, 0.24 dB, 0.23 dB, and 0.03 dB. Meanwhile, on average, the proposed MS-GSRC achieve an SSIM improvement of 0.0153 on BM3D, 0.0169 on PGPD, 0.0064 on WNNM, 0.0137 on NCSR, 0.0228 on RRC, 0.0027 on LGSR, and 0.0194 on GSRC-NLP. Evidently, our proposed MS-GSRC method yields better PSNR and SSIM in almost all noise cases. Our method is only 0.01 dB lower than WWNM in PSNR, but 0.0052 higher than in SSIM at *σ* = 75. Beyond objective metrics, the subjective perception of the human body is also a crucial criterion for assessing the quality of an image. Consequently, we present the visual contrast between the two images of starfish and 223,061 restored by different methods in Figures 3, 4, respectively. Figure 3 indicates that BM3D, PGPD, WNNM, and RRC are likely to over-smooth the restored image, whereas NCSR, GSRC-NLP, and LGSR can lead to the appearance of some undesired visual artifacts. As can be seen in Figure 4, although the image restored by WNNM has a higher PSNR, the image restored by our MS-GSRC method has a higher SSIM value and presents a better visual effect. PGPD, NCSR, RRC, and GSRC-NLP are susceptible to loss of detail in the restored images, while BM3D, WNNM, and LGSR may result in undesirable artifacts.

**Table 2:** PSNR (dB) and SSIM comparison of different methods for image denoising on BSD68 dataset.

***σ***	**15**	**30**	**50**	**75**	**Average**
BM3D	31.08	27.76	25.62	24.21	27.17
	0.8722	0.7732	0.6869	0.6221	0.7386
PGPD	31.14	27.81	25.75	24.30	27.25
	0.8697	0.7698	0.6873	0.6214	0.7370
WNNM	31.32	27.97	25.86	**24.39**	27.39
	0.8766	0.7802	0.6983	0.6348	0.7475
NCSR	31.18	27.78	25.57	24.04	27.14
	0.8769	0.7771	0.6858	0.6209	0.7402
RRC	31.07	27.74	25.67	24.18	27.17
	0.8644	0.7643	0.6840	0.6117	0.7311
LGSR	31.37	27.99	25.86	24.35	27.39
	0.8817	0.7862	0.7025	0.6347	0.7512
GSRC-NLP	31.15	27.74	25.66	24.15	27.18
	0.8681	0.7646	0.6835	0.6217	0.7345
OURS	**31.38**	**28.01**	**25.88**	24.38	**27.41**
	**0.8827**	**0.7889**	**0.7042**	**0.6400**	**0.7539**

The data marked in red represent the best values.

### 4.2. Image inpainting

Next, we verify the superiority of the MS-GSRC model on inpainting. We likewise compare the proposed MS-GSRC method with many classical or recently popular methods, such as SAIST (Afonso et al., 2010), TSLRA (Guo et al., 2017), GSR (Zhang et al., 2014b), JSM (Zhang et al., 2014c), JPG-SR (Zha et al., 2018b), LGSR (Zha et al., 2022), and IDBP (Tirer and Giryes, 2018). Among these, SAIST is one of the earliest proposed methods for image restoration, GSR, JPG-SR, LGSR, TSLRA, and JSM use the NSS prior, and IDBP is a deep learning-based method. In simulation experiments, we test images by randomly generated masks that included missing pixels of 80%, 70%, 60%, and 50%. Following are the parameters that we set for the MS-GSRC model in different cases. We set the patch size to 7 × 7, the search window size to 25, and the non-local similar patches to 60. In addition, for all cases, we set the multi-scales to [1,0.85]. Moreover, we set (0.0002, 0.0001, 1.5, 15) and (0.0001, 0.0001, 1.5, 15) as parameters (ω, ζ, *α*, *β*) when the missing pixels are 0.8 and others, respectively. In addition, $\sigma =\sqrt{2}$ for all experiences.

Table 3 illustrates the PSNR and SSIM results for each method on the 12 frequently used test images. As observed in Table 3, our proposed method exceeds the comparison algorithm virtually often when it comes to image inpainting performance. The proposed MS-GSRC outperforms SAIST, JSM, GSR, TSLRA, JPG-SR, LGSR, and IDBP approaches in average PSNR performance, with gains of 5.8 dB, 1.43 dB, 0.51 dB, 1.82 dB, 0.51 dB, 0.13 dB, and 1.26 dB, respectively. Additionally, on average, the proposed MS-GSRC surpasses SAIST by 0.0776, JSM by 0.0216, GSR by 0.0025, TSLRA by 0.0238, JPG-SR by 0.007, LGSR by 0.0012, and IDBP by 0.022.

**Table 3:** PSNR (dB) and SSIM comparison of different methods SAIST, TSLRA, GSR, JSM, JPG-SR, LGSR, IDBP, and OURS for image inpainting.

**Images**	**Bahoon**	**Bear**	**House**	**Lake**	**Leaves**	**Lena**	**Lily**	**Pepper**	**Nanna**	**Butterfly**	**Gilrs**	**Fireman**	**Average**
**Pixels missing = 80%**													
SALSA	23.15	27.29	26.63	22.20	19.78	25.96	24.31	25.55	21.96	19.95	21.80	22.17	23.40
	0.5815	0.7952	0.8421	0.7420	0.7749	0.8294	0.7485	0.8633	0.7288	0.7883	0.7078	0.6812	0.7569
JSM	25.21	29.35	34.28	25.57	26.17	30.50	27.92	30.26	25.16	25.38	25.07	25.25	27.51
	0.6577	0.8378	0.9102	0.8302	0.9209	0.8991	0.8410	0.9214	0.8196	0.9011	0.8015	0.7664	0.8423
GSR	24.58	30.28	**35.57**	25.67	27.46	31.42	28.87	31.10	25.23	26.03	25.50	25.46	28.10
	0.6893	0.8650	0.9313	0.8560	**0.9452**	0.9250	0.8820	0.9393	0.8531	0.9223	0.8386	0.8041	0.8709
TSLRA	**25.44**	29.34	31.30	25.31	25.09	30.09	27.96	28.39	25.32	24.91	24.99	25.44	26.96
	0.6714	0.8401	0.9106	0.8103	0.8934	0.8904	0.8400	0.9087	0.8163	0.8835	0.7974	0.7759	0.8365
JPG-SR	24.99	30.15	34.92	25.93	27.42	31.46	28.97	31.23	25.66	26.29	25.60	25.48	28.18
	0.6904	0.8562	0.9148	0.8508	0.9409	0.9193	0.8767	0.9326	0.8500	0.9214	0.8373	0.7977	0.8657
LGSR	25.24	30.55	35.83	26.33	27.48	31.69	29.07	31.75	25.91	26.53	25.81	25.79	28.50
	0.6989	0.8678	**0.9333**	0.8611	0.9419	0.9251	0.8813	0.9383	0.8541	0.9244	0.8423	0.8078	0.8730
IDBP	25.03	30.06	33.69	25.84	26.48	30.29	28.10	30.89	25.42	25.60	25.48	25.46	27.70
	0.6695	0.8447	0.9060	0.8319	0.9233	0.8979	0.8486	0.9153	0.8214	0.9011	0.8146	0.7645	0.8449
OURS	25.32	**30.62**	35.55	**26.38**	**27.60**	**31.91**	**29.20**	**31.97**	**26.06**	**26.78**	**25.97**	**26.04**	**28.62**
	**0.7006**	0.8694	**0.9246**	**0.8619**	**0.9436**	**0.9267**	**0.8840**	**0.9405**	**0.8564**	**0.9278**	**0.8461**	**0.8125**	**0.8745**
**Pixels missing = 70%**													
SALSA	24.32	29.29	27.49	24.33	22.01	28.10	26.20	28.40	23.93	22.41	23.53	23.96	25.33
	0.6867	0.8542	0.8827	0.8325	0.8572	0.8864	0.8278	0.9159	0.8179	0.8669	0.7962	0.7703	0.8329
JSM	26.48	31.56	36.69	27.56	29.28	32.67	29.74	33.28	27.19	27.84	27.18	27.07	29.71
	0.7514	0.8895	0.9402	0.8854	0.9581	0.9351	0.8924	0.9535	0.8819	0.9374	0.8739	0.8385	0.8948
GSR	26.17	32.01	37.63	28.08	31.18	33.54	31.10	34.77	27.89	28.92	27.86	27.47	30.55
	0.7797	0.9043	0.9543	0.9057	**0.9744**	0.9507	0.9246	**0.9633**	0.9076	0.9506	0.9015	0.8681	0.9154
TSLRA	26.71	31.65	35.86	27.30	27.94	32.58	29.91	32.64	27.27	27.74	27.05	27.23	29.49
	0.7602	0.8917	0.9485	0.8770	0.9440	0.9355	0.8942	0.9494	0.8808	0.9342	0.8668	0.8412	0.8936
JPG-SR	26.38	32.21	37.41	28.04	30.89	33.58	31.12	34.49	27.95	29.18	27.91	27.54	30.56
	0.7774	0.8997	0.9445	0.9011	0.9707	0.9469	0.9197	0.9580	0.9036	0.9494	0.8982	0.8624	0.9110
LGSR	26.65	32.28	**37.98**	28.72	31.31	33.76	31.19	34.92	28.21	29.39	28.14	27.90	30.87
	0.7846	0.9065	**0.9555**	0.9097	0.9729	0.9507	0.9237	0.9619	0.9065	0.9523	0.9025	0.8707	0.9165
IDBP	26.39	31.74	36.48	27.92	29.23	32.58	30.08	33.36	27.16	28.25	27.49	27.37	29.84
	0.7582	0.8872	0.9293	0.8856	0.9549	0.9340	0.8974	0.9460	0.8767	0.9387	0.8766	0.8391	0.8936
OURS	**26.76**	**32.38**	37.97	**28.77**	**31.57**	**33.88**	**31.53**	**35.11**	**28.42**	**29.62**	**28.35**	**28.13**	**31.04**
	**0.7867**	**0.9078**	0.9541	**0.9102**	**0.9744**	**0.9515**	**0.9284**	0.9632	**0.9090**	**0.9542**	**0.9053**	**0.8743**	**0.9183**
**Pixels missing = 60%**													
SALSA	25.40	29.73	29.99	25.84	24.65	29.69	28.11	30.60	25.37	25.28	25.06	25.37	27.09
	0.7648	0.8880	0.9096	0.8772	0.9192	0.9203	0.8848	0.9443	0.8688	0.9186	0.8536	0.8349	0.8820
JSM	27.71	33.07	38.53	29.35	31.43	34.60	31.56	35.35	29.06	29.77	28.96	28.72	31.51
	0.8175	0.9182	0.9580	0.9213	0.9748	0.9559	0.9278	0.9678	0.9182	0.9567	0.9151	0.8871	0.9265
GSR	27.74	33.60	39.68	29.86	33.39	35.81	33.05	36.42	30.13	31.09	29.55	29.32	32.47
	0.8445	0.9298	0.9674	0.9366	0.9849	0.9668	0.9505	0.9739	0.9383	0.9667	0.9359	0.9086	0.9420
TSLRA	27.92	32.77	37.23	29.01	30.19	34.26	31.55	34.96	29.17	29.42	28.79	28.73	31.17
	0.8239	0.9195	0.9641	0.9156	0.9666	0.9555	0.9282	0.9654	0.9173	0.9531	0.9097	0.8860	0.9254
JPG-SR	27.92	33.61	39.22	30.13	33.26	35.73	33.10	36.40	30.21	31.30	29.84	29.46	32.52
	0.8404	0.9240	0.9594	0.9328	0.9829	0.9626	0.9464	0.9692	0.9350	0.9641	0.9326	0.9039	0.9378
LGSR	28.15	33.94	39.82	30.66	33.70	35.97	33.31	36.85	30.40	31.58	30.13	29.83	32.86
	0.8481	0.9320	**0.9678**	0.9396	0.9848	0.9665	0.9506	0.9732	0.9381	0.9673	0.9373	0.9119	0.9431
IDBP	27.71	33.53	38.18	29.76	31.55	34.35	31.85	35.27	29.22	29.71	29.24	28.98	31.61
	0.8226	0.9176	0.9487	0.9209	0.9728	0.9531	0.9301	0.9628	0.9184	0.9544	0.9153	0.8839	0.9251
OURS	**28.23**	**34.10**	**39.90**	**30.71**	**34.11**	**36.08**	**33.54**	**36.99**	**30.55**	**31.84**	**30.43**	**30.05**	**33.04**
	**0.8481**	**0.9331**	0.9676	**0.9402**	**0.9861**	**0.9672**	**0.9528**	**0.9740**	**0.9396**	**0.9684**	**0.9395**	**0.9142**	**0.9442**
**Pixels missing = 50%**													
SALSA	26.50	31.79	31.64	27.83	26.61	30.98	29.59	31.08	26.85	27.28	26.90	27.09	28.68
	0.8270	0.9226	0.9326	0.9176	0.9471	0.9436	0.9181	0.9595	0.9062	0.9452	0.8992	0.8826	0.9168
JSM	29.05	34.63	40.43	30.99	33.80	36.37	33.41	37.32	30.73	31.35	30.63	30.27	33.25
	0.8697	0.9415	0.9710	0.9447	0.9848	0.9705	0.9523	0.9773	0.9440	0.9692	0.9433	0.9196	0.9490
GSR	29.41	35.62	41.62	32.14	35.87	37.63	35.41	38.53	32.16	32.78	31.93	31.00	34.51
	0.8923	0.9509	0.9768	0.9575	0.9909	0.9779	0.9685	**0.9817**	0.9589	0.9759	0.9582	0.9353	0.9604
TSLRA	29.15	33.01	40.22	30.53	32.56	35.52	33.20	36.61	30.87	31.01	30.48	30.25	32.79
	0.8734	0.9407	0.9748	0.9409	0.9803	0.9702	0.9518	0.9758	0.9433	0.9672	0.9397	0.9186	0.9480
JPG-SR	29.49	35.53	40.85	31.89	35.83	37.39	35.21	38.19	32.27	32.89	32.02	30.96	34.38
	0.8887	0.9454	0.9704	0.9533	0.9896	0.9732	0.9647	0.9771	0.9558	0.9737	0.9556	0.9310	0.9565
LGSR	29.74	35.89	41.78	32.57	36.35	37.89	35.41	38.59	32.50	33.38	32.19	31.42	34.81
	0.8950	0.9524	**0.9772**	0.9592	0.9910	0.9775	0.9684	0.9810	0.9592	0.9771	0.9592	0.9379	0.9613
IDBP	29.14	34.85	40.20	31.51	34.05	36.36	33.66	37.60	30.86	31.99	31.11	30.53	33.49
	0.8726	0.9383	0.9653	0.9447	0.9836	0.9668	0.9523	0.9738	0.9420	0.9686	0.9427	0.9170	0.9473
OURS	**29.80**	**35.99**	**41.80**	**32.58**	**36.60**	**38.07**	**35.63**	**38.89**	**32.53**	**33.44**	**32.38**	**31.58**	**34.94**
	**0.8950**	**0.9530**	0.9769	**0.9595**	**0.9915**	**0.9780**	**0.9694**	**0.9817**	**0.9597**	**0.9775**	**0.9604**	**0.9395**	**0.9618**

The data marked in red represent the best values.

**Figure 5:**
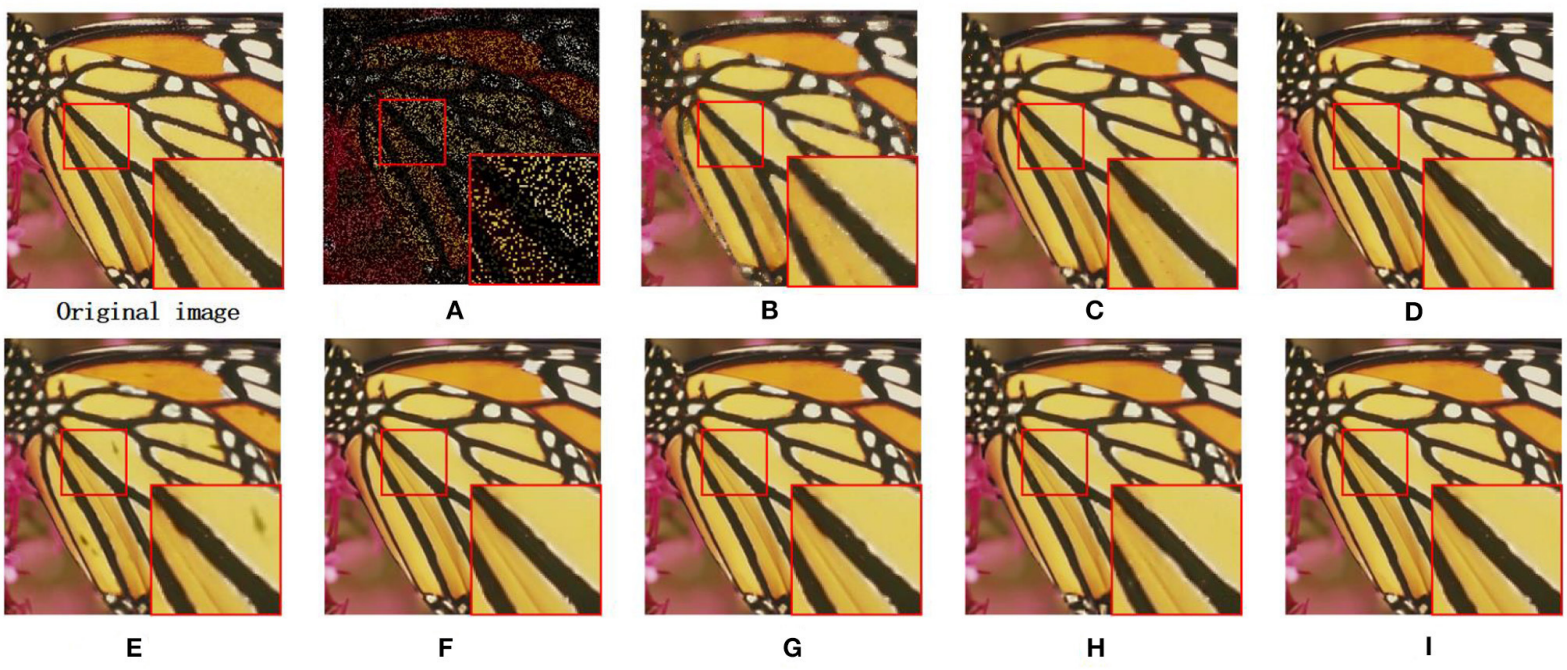
Inpainting results on image butterfly (missing ratio=80%). **(A)** Missing pixels image. **(B)** SAIST(PSNR = 19.95 dB and SSIM = 0.7883). **(C)** JSM (PSNR = 25.38 dB and SSIM = 0.9011). **(D)** GSR (PSNR = 26.03 dB and SSIM = 0.9223). **(E)** TSLRA (PSNR = 24.91 dB and SSIM = 0.8835). **(F)** JPG-SR (PSNR = 26.29 dB and SSIM = 0.9214). **(G)** LGSR (PSNR = 26.53 dB and SSIM = 0.9244). **(H)** IDBP (PSNR = 25.60 dB and SSIM = 0.9011). **(I)** OURS(PSNR = **26.78** dB and SSIM = **0.9278**).

**Figure 6:**
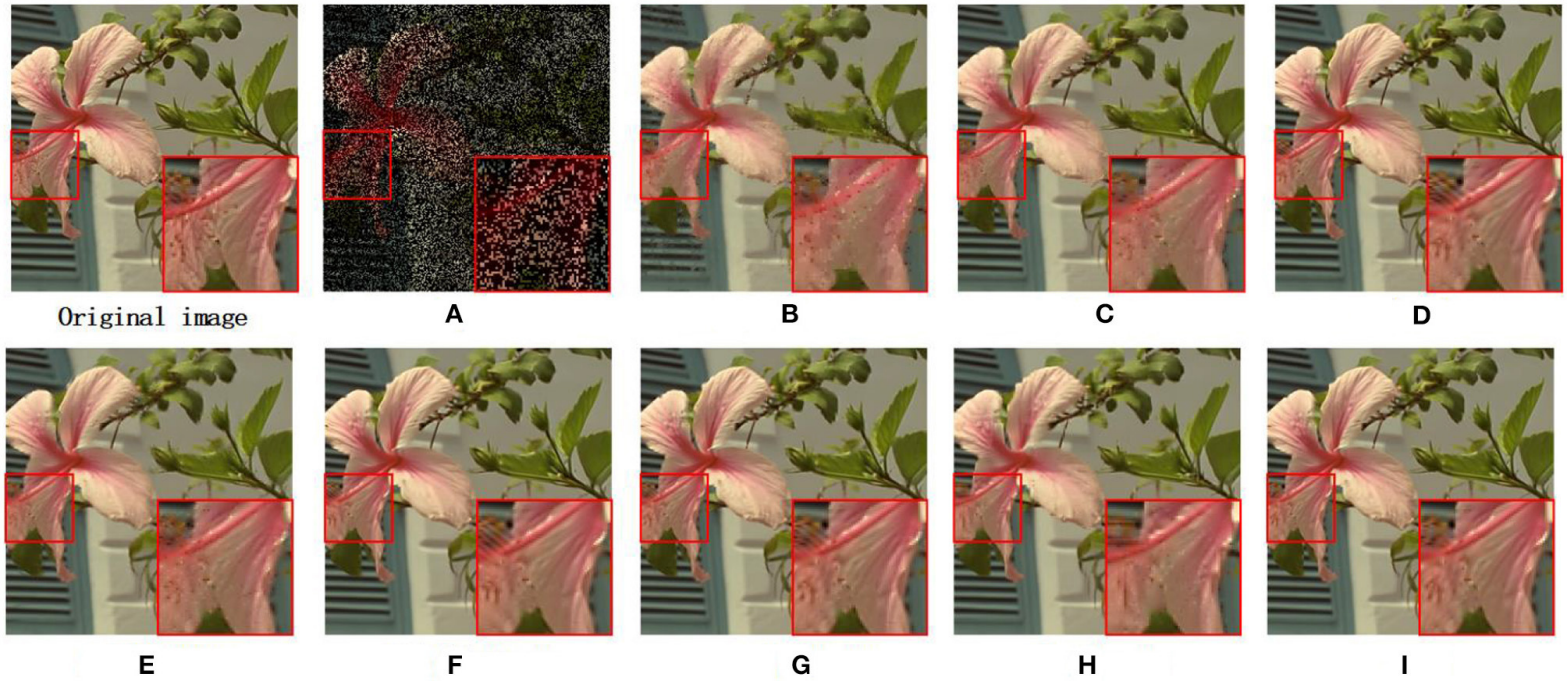
Inpainting results on image flowers (missing ratio = 70%). **(A)** Missing pixels image. **(B)** SAIST (PSNR = 27.69 dB and SSIM = 0.8422). **(C)** JSM (PSNR = 29.74 dB and SSIM =0.8924). **(D)** GSR (PSNR = 31.10 dB and SSIM = 0.9246). **(E)** TSLRA (PSNR = 29.91 dB and SSIM = 0.8942). **(F)** JPG-SR (PSNR = 31.12 dB and SSIM = 0.9197). **(G)** LGSR (PSNR = 31.19 dB and SSIM = 0.9237). **(H)** IDBP (PSNR = 30.08 dB and SSIM = 0.8974). **(I)** OURS (PSNR = **31.53** dB and SSIM = **0.9284**).

Similarly, two images are selected for detailed visual analysis. The image butterfly with a 80% loss of pixels restored by different methods are presented in Figure 5. Moreover, Figure 6 displays the outcomes of a visual comparison of image flowers with a 70% loss of pixels restored with different algorithms. By analyzing the visual comparison images, we can find that images restored using SAIST, JSM, TSLRA, IDBP GSR, and JPG-SR are susceptible to excessive smoothing, and images restored using LGSR tend to show excessive visual artifacts. The images restored using our proposed MS-GSRC model have significantly better restoration capabilities with regard to image detail and edges.

### 4.3. Image compressed sensing

Finally, we validate the restoration capability of our proposed MS-GSRC model on the image compressed sensing problem. In this part of experiments, we use the Gaussian random projection matrix (Zhang et al., 2014b) to generate blocks of size 32 × 32 to test the CS restoration effects. The parameters set for the MS-GSRC model are as follows: For all cases, the patch size is set to be 8 × 8, the patch number to 80, the search window size to be 25, and the multi-scales to be [1,0.75]. In addition, (0.004, 0.00002, 0.6, 25), (0.0014, 0.00005, 0.9, 15), (0.0015, 0.00001, 0.5, 10), and (0.0015, 0.00001, 1.4, 6) are set for (ζ,ω, *α*,*β*) when subrate is 0.1N, 0.2N, 0.3N, and 0.4N.

BSC (Mun and Fowler, 2009), RCOS (Zhang et al., 2012), ALSB (Zhang et al., 2014a), GSR (Zhang et al., 2014b), ASNR (Zha et al., 2018a), and LGSR (Zha et al., 2022) are choosen as competing methods. Among them, GSR performs a sparse representation on similar groups of images, ASNR is an image of the CS method that extends on the basis of NCSR, and LGSR combines sparsity and LR. Similarly, we selected 12 images frequently used in image restoration experiments as test images. Table 4 presents the average outcomes of PSNR and SSIM of the restored images using different method. To be concrete, the proposed MS-GSRC model over BCS, RCOS, ALSB, GSR, ASNR, and LGSR methods are 5.36 dB, 2.34 dB, 1.24 dB, 0.46 dB, 0.45 dB, and 0.14d B in PSNR and 0.1591, 0.0634, 0.0023, 0.0056, 0.0082, and 0.0029 in SSIM, respectively.

**Table 4:** PSNR (dB) and SSIM comparison of different methods for image CS on 12 test images.

**Subrate**	**0.1**	**0.2**	**0.3**	**0.4**	**Average**
BCS	23.60	26.26	28.19	29.88	26.98
	0.6308	0.7418	0.8117	0.8609	0.7445
RCOS	25.92	29.20	31.54	33.34	30.00
	0.7163	0.8298	0.8909	0.9236	0.8402
ALSB	26.66	30.19	32.67	34.87	31.10
	0.7778	0.8751	0.9209	0.9484	0.8806
GSR	27.00	30.96	33.66	35.89	31.88
	0.8002	0.8963	0.9367	0.9587	0.8980
ASNR	27.24	31.04	33.51	35.78	31.89
	0.7965	0.8953	0.9329	0.9568	0.8954
LGSR	27.51	31.34	33.89	36.07	32.20
	0.8062	0.8994	0.9379	0.9593	0.9007
OURS	**27.91**	**31.40**	**33.94**	**36.09**	**32.34**
	**0.8150**	**0.9009**	**0.9387**	**0.9598**	**0.9036**

The data marked in red represent the best values.

**Figure 7:**
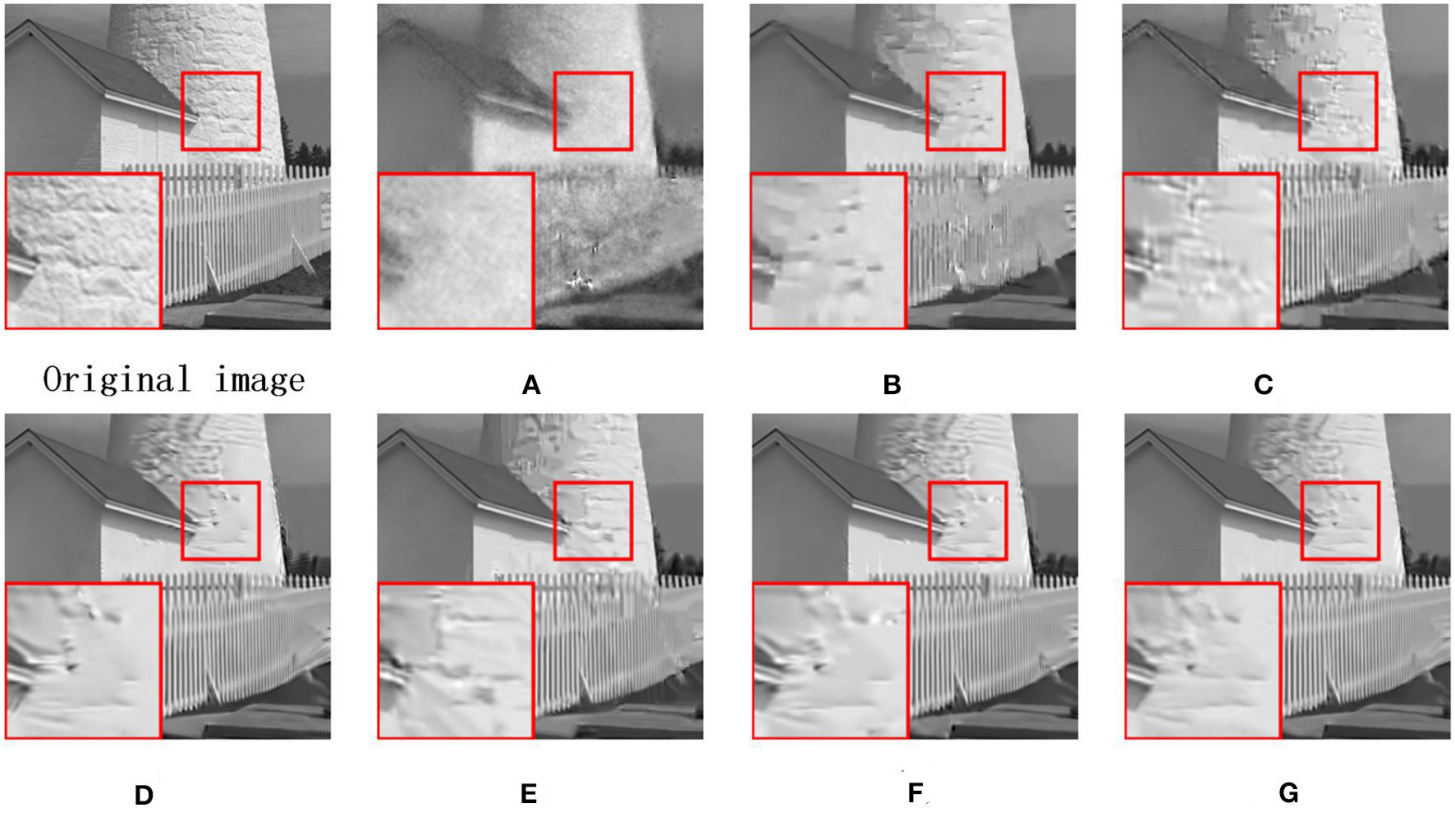
CS results on image fence (subrate = 0.1 N). **(A)** BCS (PSNR = 19.54 dB, SSIM = 0.5034). **(B)** RCOS (PSNR = 23.29 dB, SSIM = 0.6932). **(C)** ALSB (PSNR = 25.05 dB and SSIM = 0.7736). **(D)** GSR (PSNR = 26.06 dB and SSIM = 0.8047). **(E)** ASNR (PSNR = 26.01 dB and SSIM = 0.8006). **(F)** LGSR (PSNR = 26.58 dB and SSIM = 0.8107). **(G)** OURS (PSNR = **27.26** dB and SSIM = **0.8216**).

**Figure 8:**
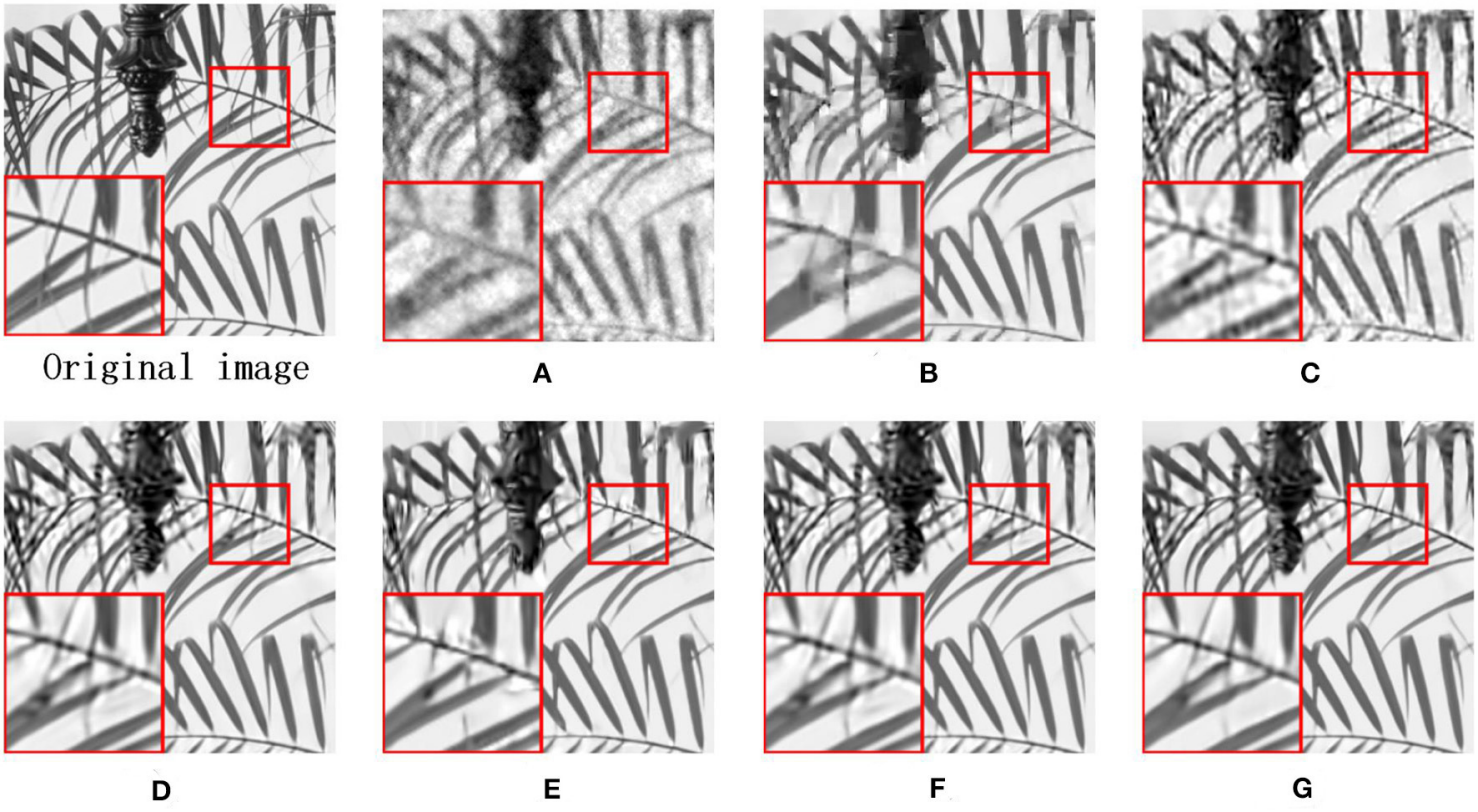
CS results on image leaves (subrate = 0.1 N). **(A)** BCS (PSNR = 18.37 dB, SSIM = 0.5767). **(B)** RCOS (PSNR = 22.17 dB, SSIM = 0.0.8323). **(C)** ALSB (PSNR = 21.52 dB and SSIM = 0.7939). **(D)** GSR (PSNR = 23.22 dB and SSIM = 0.8731). **(E)** ASNR (PSNR = 23.48 dB and SSIM = 0.8805). **(F)** LGSR (PSNR = 23.75 dB and SSIM = 0.8824). **(G)** OURS (PSNR = **24.57** dB and SSIM = **0.8992**).

**Figure 9:**
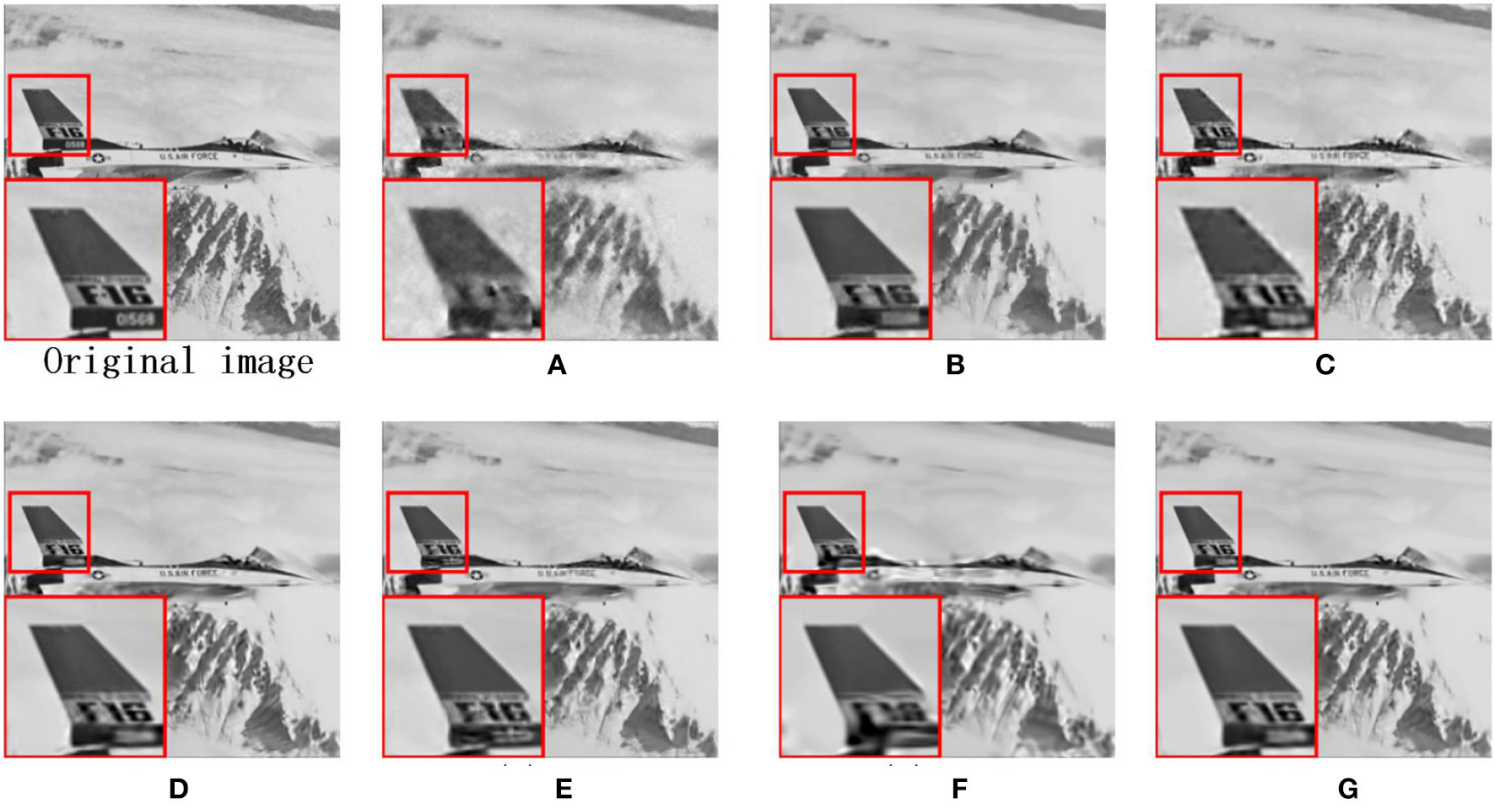
CS results on image airplane (subrate = 0.2 N). **(A)** BCS (PSNR = 25.87 dB, SSIM = 0.8111). **(B)** RCOS (PSNR = 28.22 dB, SSIM = 0.8854). **(C)** ALSB (PSNR = 28.39 dB and SSIM = 0.8942). **(D)** GSR (PSNR = 28.87 dB and SSIM = 0.9082). **(E)** ASNR (PSNR = 29.17 dB and SSIM = 0.9075). **(F)** LGSR (PSNR = 29.43 dB and SSIM = 0.9110). **(G)** OURS (PSNR = **29.59** dB and SSIM = **0.9120**).

Due to the other competing algorithms used in this thesis, all use BCS to pre-process CS images, and here we use the BCS-processed images as corrupted images. Figure 7 shows the visual contrast of the image fence with 0.1 N CS measurements, and we can observe that RCOS and ALSB are less capable of restoring details, GSR and LGSR lead to over-smooth, and ASNR generates some redundant artifacts. Figure 8 illustrates the visual comparison of the image leaves measured with 0.1N CS. All comparison images have strong ringing phenomena and present terrible artifacts. In Figure 9, we have selected the image airplane processed with 0.2N CS for detailed analysis. It is obvious that the details of the images restored by ALSB and LGSR are seriously missing. The images restored by RCOS, GSR, and ASNR produced more artifacts. In the above three cases, our proposed MS-GSRC algorithm significantly outperforms other competing algorithms in recovering the image overall and some texture details.

## 5. Conclusion

In this study, we propose a novel model Multi-Scale Group Sparse Residual Constraint Model (MS-GSRC) for image restoration. This model introduces the low-rank property into the group sparse residual framework and finds similar patches for overlapping patches of the input image using a multi-scale strategy. Furthermore, under the MAP restoration framework, an alternating minimization method with adaptive tunable parameters is used to deliver a robust optimization solution for our MS-GSRC method. We employ the MS-GSRC model to three image restoration problems, namely, denoising, inpainting, and compressed sensing. Extensive simulation trials show that our novel model performs superior to many classical methods in terms of both objective image quality and subjective visual quality.

## Data availability statement

The raw data supporting the conclusions of this article will be made available by the authors, without undue reservation.

## Author contributions

WN: Writing — original draft. DS: Writing — review & editing. QG: Writing — review & editing. YL: Writing — review & editing. DZ: Writing — review & editing.
